# adaPop: Bayesian inference of dependent population dynamics in coalescent models

**DOI:** 10.1371/journal.pcbi.1010897

**Published:** 2023-03-20

**Authors:** Lorenzo Cappello, Jaehee Kim, Julia A. Palacios

**Affiliations:** 1 Departments of Economics and Business, Universitat Pompeu Fabra, Barcelona, Spain; 2 Department of Computational Biology, Cornell University, Ithaca, New York, United States of America; 3 Departments of Statistics and Biomedical Data Science, Stanford University, Stanford, California, United States of America; The University of Melbourne Melbourne School of Psychological Sciences, AUSTRALIA

## Abstract

The coalescent is a powerful statistical framework that allows us to infer past population dynamics leveraging the ancestral relationships reconstructed from sampled molecular sequence data. In many biomedical applications, such as in the study of infectious diseases, cell development, and tumorgenesis, several distinct populations share evolutionary history and therefore become dependent. The inference of such dependence is a highly important, yet a challenging problem. With advances in sequencing technologies, we are well positioned to exploit the wealth of high-resolution biological data for tackling this problem. Here, we present adaPop, a probabilistic model to estimate past population dynamics of dependent populations and to quantify their degree of dependence. An essential feature of our approach is the ability to track the time-varying association between the populations while making minimal assumptions on their functional shapes via Markov random field priors. We provide nonparametric estimators, extensions of our base model that integrate multiple data sources, and fast scalable inference algorithms. We test our method using simulated data under various dependent population histories and demonstrate the utility of our model in shedding light on evolutionary histories of different variants of SARS-CoV-2.

## Author summary

Genomic data provide information about evolutionary dynamics—such as an evolving epidemic and tumorgenesis—that is difficult to infer from other source of data. One of the main computational challenges in inferring past population histories is to jointly model dependent subpopulations and correctly quantifying their dependencies over time. When distinct subpopulations have common ancestry in the past and evolve under shared environmental pressure, their population dynamics become dependent. In this work, we propose an efficient inference method for studying dependent population dynamics from genetic data in the coalescent framework: an approach that considers the stochastic process of the “coalescence” of genealogical lineages traveling back in time to explain the statistical properties of a sample’s genetic variation. We also extend our framework to jointly model the ancestral and sampling processes incorporating sampling times as an additional source of information. We validate our methods via extensive simulations and demonstrate that our methods provide new insights into the evolutionary dynamics of SARS-CoV-2 novel variants.

This is a *PLOS Computational Biology* Methods paper.

## Introduction

Bayesian inference of past population sizes from genetic data is an important task in molecular epidemiology of infectious diseases and other biomedical disciplines [1–4]. While many computational and methodological advances have been developed in the last 20 years, there are still many challenges in using these models on real data applications (see [5, 6] for recent reviews).

One of the important limitations of most current methods is their lack of flexibility in modeling the dependency of populations’ effective population sizes. Current models typically assume either single population size dynamics or a structured population undergoing migration. In particular, when modeling structured populations, these models resort to simplistic assumptions on the population size dynamics in order to gain computational tractability and parameter identifiability [7, 8]. However, often there are situations where the population is neither one large unit nor completely divided into isolated subpopulations. Different subpopulations may share the same environment and partial ancestry some time in the past, and therefore their population dynamics are dependent. For example, in the study of infectious diseases, all variants of a virus may share the same environment and local non-pharmaceutical interventions; hence their population dynamics could be similarly affected by these external interventions. In tumorgenesis, the cancer cell population within an individual undergoes clonal expansions in the tumor microenvironment, often resulting in a mixture of genotypically and phenotypically heterogenous cell subpopulations. Identifying and quantifying such expansions is crucial for timely detection and personalized oncology for cancer [9]. Despite its importance, to the best of our knowledge, no realistic methods exist for jointly modeling and studying the trajectories of dependent population size trajectories.

In this work, we propose a nonparametric method for inferring dependent past population dynamics of subpopulations and for estimating the relative difference in their population size trajectories over time. The proposed method bypasses the problems inherent in modeling complex dependent population dynamics by *a priori* modeling the dependency of population sizes via a nonparametric prior. Our method not only provides a measure of this dependence, but also increases estimates’ accuracy and generates narrower credible bands. Essentially, this happens because we can employ more data to estimate the parameter of interest. In addition, our approach incorporates other types of data informative of the parameter of interest such as temporal sampling information of molecular sequences.

Our contributions can be summarized as follows.

- We propose a nonparametric Bayesian framework for inferring dependent population dynamics from genetic data in the coalescent framework. The model makes minimal assumptions on the functional form of the population trajectories and their dependency. Despite its flexibility, we prove that model parameters are identifiable.
- We extend our framework to jointly model the ancestral and sampling processes incorporating sampling times as an additional source of information. We empirically validate the performance of our methods and show the ability of the sampling-aware methods in reducing bias and improving estimation.
- We demonstrate the utility of our methods in providing new insights into the evolutionary dynamics of SARS-CoV-2 novel variants.

## Background

### Coalescent model

**Fig 1:**
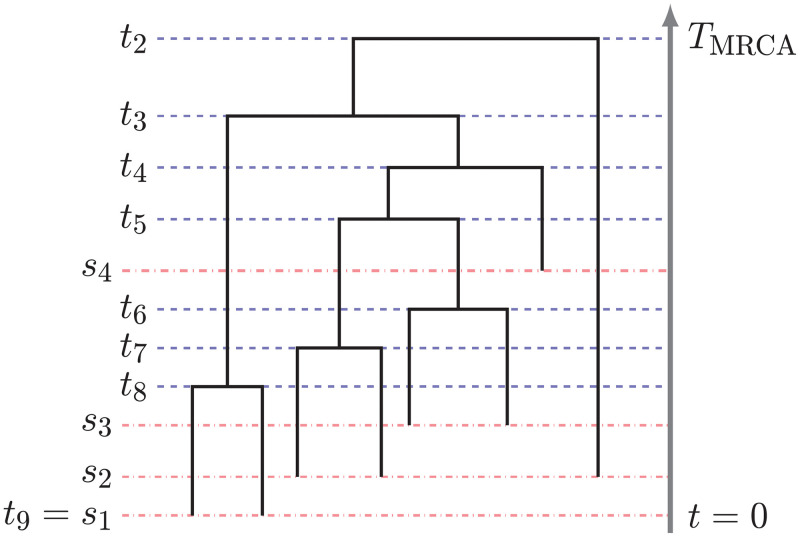
Example of a genealogy with sequential sampling. *s*_1_, …, *s*_4_ and *t*_8_, …, *t*_2_ indicate sampling times (red dotted lines) and coalescent times (blue dotted lines), respectively. The time increases backwards in time starting with *s*_1_ = *t*_9_ = 0 as the present time. *T*_MRCA_ indicates the time to the most recent common ancestor at the root.

The coalescent [10] is a popular prior model on genealogies. The genealogy is a timed and rooted binary tree that represents the ancestry of a sample of *n* individuals from a population. We assume that *g* is a discrete ranked and labeled tree topology with *n* leaves, (*n*_*ℓ*_)_*ℓ*=1:*m*_ samples are sequentially collected at *m* sampling times denoted by **s** = (*s*_*ℓ*_)_*ℓ*=1:*m*_, with *s*_1_ = 0, *s*_*j*−1_ < *s*_*j*_ for *j* = 2, …, *m*, and $n=\sum_{j=1}^{m}n_{j}$ is the total number of samples. In the genealogy, pairs of lineages merge backwards in time into a common ancestor at coalescent times denoted by **t** = (*t*_*n*_, …, *t*_2_) (Fig 1). The rate at which pairs of lineages coalesce depends on the number of lineages and the effective population size (EPS) denoted by (*N*_*e*_(*t*))_*t* ≥ 0_ ≔ *N*_*e*_. Under this model, the density of a genealogy **g** = (*g*, **t**, **s**, **n**) is:

$$\begin{array}{c} p\left(g|N_{e}\right)=\text{exp}(-\int_{0}^{\infty }\frac{C(t)}{N_{e}\left(t\right)}dt)\prod_{k=2}^{n}\frac{1}{N_{e}\left(t_{k}\right)}, \end{array}$$ (1)

where $C\left(t\right)=\left(\begin{array}{c} A\left(t\right)\\ 2 \end{array}\right)$ is the coalescent factor—a combinatorial factor of the number of extant lineages $A\left(t\right)=\sum_{i=1}^{m}n_{i}1\left(s_{i}<t\right)-\sum_{k=2}^{n}1\left(t_{k}<t\right)$. The EPS is generally interpreted as a relative measure of genetic diversity [11].

### Bayesian inference of EPS

We start by assuming that a given genealogy is available to us. Bayesian inference of the EPS then targets

$$\begin{array}{c} P\left(N_{e},\tau |g\right)\propto P\left(g|N_{e}\right)P\left(N_{e}|\tau \right)P\left(\tau \right), \end{array}$$ (2)

where *P*(*N*_*e*_∣***τ***) is a prior distribution on *N*_*e*_ that depends on a vector of hyperparameters ***τ***.

A common choice for a prior on *N*_*e*_ is to assume an underlying finite-dimensional parametric structure. In particular, this choice makes the calculation of the integral in Eq (1) computationally tractable. A popular strategy is to use a regular grid of *M* + 1 points (*k*_*i*_)_1:*M*+1_ and assume that *N*_*e*_ is well approximated by

$$\begin{array}{c} N_{e}\left(t\right)=\sum_{i=1}^{M+1}\text{exp}\left(\theta_{i}\right)1\left(t\in \left(k_{i},k_{i+1}\right)\right), \end{array}$$ (3)

a piece-wise constant function with *M* change points [1, 12]. In Eq (3), we model *N*_*e*_ in log-scale, however this is not strictly necessary. Many approaches place a Gaussian Markov random field (GMRF) prior on ***θ***, for example [13–16]. An alternative to the piece-wise constant assumption is to use Gaussian process priors. However, the posterior distribution becomes doubly intractable because the likelihood function depends on an infinite-dimensional integral over Gaussian processes. In [17], the authors augmented the posterior distribution with auxiliary variables via thinning of Poisson processes [18] in order to gain tractability.

The advantage of the GP prior is that one does not need to specify a grid. This comes at the additional computational costs mentioned above. GMRFs priors require selecting a grid; however, current formulations (e.g., [16]) leave ample flexibility in the choice of the number of parameters *M* and grid breakpoints while remaining computationally extremely tractable. This is the case because neither the grid cell boundaries (*k*_*i*_)_1:*M*+1_ nor *M* depend on the data **g** = (*g*, **t**, **s**, **n**) or the model parameters *N*_*e*_(*t*). By increasing *M*, one effectively enriches the family of functions supported by the prior, which is the motivation underlying the use of a GP. We refer to [16] for guidance on the choice of *M*.

There are exact and approximate algorithms to sample from Eq (2). Standard software packages [3, 19] employ Markov chain Monte Carlo (MCMC) methods with carefully designed transition kernels. Recent algorithmic advances employ Hamiltonian Monte Carlo (HMC). [16] implemented an algorithm to sample from Eq (2) under several Markov random fields priors on STAN [20], and Lan et al. [21] employed split HMC [22]. Among the approximate methods, inference can be efficiently done with Integrated Nested Laplace Approximation (INLA) [23]. In [24], authors use INLA under a GMRF prior on *N*_*e*_, showing that the approximate posterior is remarkably similar to that obtained by MCMC-based algorithms.

### Preferential sampling

The standard coalescent model implicitly assumes that the sampling times are either fixed or functionally independent of the underlying population dynamics. This assumption is in stark contrast with birth-death-sampling models, where one needs to specify a sampling process along with the evolutionary model [25]. However, in many applications, such as in infectious diseases, the sampling frequency is often highly correlated with EPS: more samples are sequenced when the EPS is larger. This situation, known as preferential sampling in spatial statistics [26], allows us to model sampling frequency information in order to improve inference about EPS, reducing estimation bias and improving the accuracy of model parameter inference. A parametric preferential sampling model was first introduced in coalescent inference by [25] and later extended to the nonparametric setting [27–30]. The probabilistic dependency of sampling time distribution on population dynamics can be modeled as an inhomogeneous Poisson point process (iPPP) with a rate *λ*(*t*) that depends on EPS. Although in practice, sampling events occur in bulk, we assume that samples arrive at an instantaneous rate within a time interval. Hence, we approximate the likelihood of sampling events by the counting process over a fixed grid.

We adopt the adaptive preferential sampling framework [30] that employs a flexible approach for modeling the time-varying dependency between *N*_*e*_(*t*) and *λ*(*t*): *λ*(*t*) = *ζ*(*t*)*N*_*e*_(*t*), where both *ζ*(*t*) and *N*_*e*_(*t*) are unknown continuous functions with GMRF priors.

## Methods

We propose a flexible and scalable framework for modeling the genealogies of two samples (not necessarily of the same size) from two populations with dependent population size dynamics. The goals are (i) to estimate the EPSs of the two populations, (ii) to account for the dependency between them, and (iii) to quantify estimation uncertainty. The implementation of our methods adaPop is available in the R package adapref (https://github.com/lorenzocapp/adapref).

### Coalescent for dependent population size dynamics

Let **g**^*A*^ = (*g*^*A*^, **t**^*A*^, **s**^*A*^, **n**^*A*^) and **g**^*B*^ = (*g*^*B*^, **t**^*B*^, **s**^*B*^, **n**^*B*^) be the genealogies of samples collected from populations *A* and *B* respectively, and let $N_{e}^{A}$ and $N_{e}^{B}$ denote their corresponding EPSs. Here, (**s**^*A*^, **s**^*B*^) are the vectors of sampling times, and (**n**^*A*^, **n**^*B*^) are the corresponding vectors of number of samples collected. Standard coalescent-based inference methodologies ignore any association between the underlying population processes of the two populations when approximating posterior distributions $P(N_{e}^{A}|g^{A})$ and $P(N_{e}^{B}|g^{B})$.

The advantages of directly modeling the dependence are twofold. First, we get a direct measure of the association that can have a direct interpretation in scientific studies. Second, our hierarchical model should estimate *N*_*e*_ more accurately because we model *N*_*e*_ as a shared parameter, hence we borrow information from the two samples, which is a standard advantage of a hierarchical Bayesian model [31].

We model the association between the two population size trajectories, which can change over time, with a time-varying parameter linking $N_{e}^{A}$ and $N_{e}^{B}$:

$$\begin{array}{c} \begin{array}{ccc} g^{A}|N_{e},s^{A},n^{A} & ∼ & \text{Coalescent}\;(N_{e}),\\ g^{B}|N_{e},\gamma ,s^{B},n^{B} & ∼ & \text{Coalescent}\;(\gamma \;N_{e}),\\ \text{log}\;N_{e} & ∼ & \text{GMRF}(\tau_{1}),\\ \tau_{1}|a,b & ∼ & \text{Gamma}(a,b),\\ \text{log}\;\gamma |\tau_{2} & ∼ & \text{GMRF}(\tau_{2}),\\ \tau_{2}|a,b & ∼ & \text{Gamma}(a,b). \end{array} \end{array}$$ (4)

Here, *γ* ≔ (*γ*(*t*))_*t* ≥ 0_ is the time-varying coefficient that describes how the association between the two population processes changes over time, leading to $N_{e}^{A}=N_{e}$ and $N_{e}^{B}=\gamma N_{e}$. The interpretation of *γ* provides information on the association between two populations. For example, a growing trend in *γ* signals the existence of an association between two EPSs: $N_{e}^{B}$ is growing faster than $N_{e}^{A}$. However, it does not necessarily imply a positive association because, for example, if $N_{e}^{B}$ were growing, $N_{e}^{A}$ could be either growing at a slower rate than $N_{e}^{B}$ or be decreasing and still have an increasing *γ*.

Throughout the section, we employ GMRF priors with precision *τ*_1_ and *τ*_2_ on *N*_*e*_ and *γ*; however, the framework is flexible to any prior distribution. It is possible to go fully nonparametric employing a GP [17], or a different kind of MRFs, for example, the Horseshoe MRFs [32]. The number of parameters of the two GMRFs tunes how free the dependence is allowed to vary between the two populations. In the numerical illustrations, we will employ GMRFs modeling first order dependencies.

Our model in Eq (4) is “asymmetric”, in the sense that the baseline population EPS is multiplied by the time-varying coefficient *γ* to define the EPS of a new population. The choice is motivated by the actual scientific question we are examining, in which a new population develops from an existing one.

We will compare our proposal with a simpler parametric model suggested in [5]. Here, the population dependence is modeled through two time-independent scalar parameters *α* and *β*:

$$\begin{array}{c} N_{e}^{A}=N_{e}\;\text{and}\;N_{e}^{B}=\alpha {\left(N_{e}\right)}^{\beta }. \end{array}$$ (5)

However, the strict parametric dependence enforced in the model increases the risk of model misspecification by not allowing changes in the association of the two population processes. For example, the model support excludes a time shift when $N_{e}^{B}\left(t\right)=N_{e}^{A}\left(t+s\right)$ for *s* > 0. A consequence of this potential model misspecification is the biased estimation of $N_{e}^{B}$, *α*, and *β*; we will provide numerical proofs of this claim in Results section.

### Preferential sampling and dependent population size dynamics

A common approach for studying the relative growth between two population dynamics is to model how the sampling frequency of molecular sequences changes over time in the two populations. This is frequently done by fitting logistic growth models to the sampling dates only [33, 34]. The probabilistic models discussed in the previous section take a different stance and employ molecular data to reconstruct the genealogies which in turn are used to infer the population processes jointly. Here, we extend the probabilistic models, either model Eqs (4) or (5), and model the genealogies jointly with the observed sampling frequencies from both samples as follows:

$$\begin{array}{c} \begin{array}{ccc} s^{A}|\zeta & ∼ & \text{iPPP}(\zeta \;N_{e}^{A}),\\ s^{B}|\zeta & ∼ & \text{iPPP}(\zeta \;N_{e}^{B}),\\ \text{log}\;\zeta & ∼ & \text{GMRF}(\tau_{3}),\\ \tau_{3}|a,b & ∼ & \text{Gamma}(a,b). \end{array} \end{array}$$ (6)

Eq (6) builds on the preferential sampling framework described in Background section: the sampling process is an iPPP whose rate is a function of both the EPS and a time-varying parameter *ζ*. Here, the sampling process of populations *A* and *B* will have distinct rates, $\lambda^{A}=\zeta \;N_{e}^{A}$ and $\lambda^{B}=\zeta \;N_{e}^{B}$. Although we assumed a shared *ζ* function, our implementation considers the possibility of one *ζ* function per population. We emphasize that the model is flexible to any choice of prior distributions on *ζ*.

### Inference

We start describing the inference procedure for the parameters of the model in Eq (4). We employ the same discretization described in Background section: given a regular grid (*k*_*i*_)_1:*M*+1_, we assume that *N*_*e*_ is governed by parameters ***θ*** through the map given in Eq (3); *γ* is governed by parameters ***ξ*** = (*ξ*_*i*_)_*i* = 1:*M*′_ such that *γ*(*t*) = exp *ξ*_*i*_ for *t* ∈ (*k*_*i*_, *k*_*i*+1_]. Let ***τ*** be the vector of precision hyperparameters of the GMRFs. Following [24], we use INLA for obtaining marginal posterior medians and marginal 95% Bayesian credible intervals (BCI).

INLA does not approximate the full posterior *P*(***ξ***, ***θ***, ***τ***|**g**^*A*^, **g**^*B*^); rather, it approximates the posterior marginals *P*(***τ***|**g**^*A*^, **g**^*B*^), (*P*(*θ*_*i*_|**g**^*A*^, **g**^*B*^))_1:*M*_, and (*P*(*ξ*_*i*_|**g**^*A*^, **g**^*B*^))_1:*M*_. The first step consists in computing

$$\begin{array}{c} \hat{P}\left(\tau |g^{A},g^{B}\right)\propto \frac{P(\xi ,\theta ,\tau ,g^{A},g^{B})}{\hat{P_{G}}\left(\xi ,\theta |\tau ,g^{A},g^{B}\right)}{|}_{\begin{array}{c} \xi =\xi^{*}\left(\tau \right),\\ \theta =\theta^{*}\left(\tau \right) \end{array}}, \end{array}$$

where the denominator is the Gaussian approximation to *P*(***ξ***, ***θ***|***τ***, **g**^*A*^, **g**^*B*^) obtained from a Taylor expansion around its modes θ*(***τ***) and ξ*(***τ***) (the first Laplace approximation). The second step approximates the marginal posteriors of *P*(*θ*_*i*_|**g**^*A*^, **g**^*B*^) and *P*(*ξ*_*i*_|**g**^*A*^, **g**^*B*^). For example *P*(*θ*_*i*_|**g**^*A*^, **g**^*B*^) is approximated by

$$\begin{array}{c} \hat{P}\left(\theta_{i}|\tau ,g^{A},g^{B}\right)\propto \frac{P(\xi ,\theta ,\tau ,g^{A},g^{B})}{\hat{P_{GG}}\left(\theta_{-i},\xi |\tau ,g^{A},g^{B}\right)}{|}_{\begin{array}{c} \theta_{-i}=\theta_{-i}^{*},\\ \xi =\xi^{*}, \end{array}}, \end{array}$$

where the denominator is a further Gaussian approximation of the corresponding conditional distribution. Now the Taylor expansion is centered at (***θ***_−***i***_, *ξ*) = E_*G*_[***θ***_−***i***_, ***ξ***|***τ***, **g**^*A*^, **g**^*B*^], where the expected value is taken w.r.t. $\hat{P_{G}}\left(\theta ,\xi |\tau ,g^{A},g^{B}\right)$. The subscript *GG* highlights the fact that two Gaussian approximations are employed to define *P*_*GG*_. The last step involves integrating out the hyperparameters from $\hat{P}\left(\theta_{i}|\tau ,g^{A},g^{B}\right)$. This can be easily accomplished using $\hat{P}\left(\tau |g^{A},g^{B}\right)$ (the nested Laplace approximation step).

### Identifiability

Parameter identifiability is an essential property of models used in statistical learning. Roughly speaking, it refers to the theoretical possibility of uniquely estimating a parameter vector if an infinite amount of data is available [35–37]. Note that this is a property of the generative model, not of the estimator used.

For example, if *Y* ∼ *Poisson*(*λ*_1_*λ*_2_), then for a pair $\left(\lambda_{1}^{\prime },\lambda_{2}^{\prime }\right)$, any combination $\left(\frac{1}{c}\lambda_{1}^{\prime },c\lambda_{2}^{\prime }\right)$ with *c* > 0 will be observationally equivalent. The multiplication of two parameters is a feature often leading to unidentifiability. Despite the fact that the models described in Eqs (4) and (5) include a product of parameters, we show that identifiability is not lost.

Since the parameters of models in Eqs (4) and (5) have a scientific interpretation, a lack of identifiability could hinder the validity of the scientific insights gained from using our methodology. There is a large literature showing that many models in evolutionary biology and ecology are not identifiable [5, 38]. In the coalescent literature, [39] shows that *N*_*e*_ is identifiable in the neutral and structured case. Our proposal does not fall in these two categories and a new result is required. Under the assumption that *N*_*e*_ and *γ* are piecewise-constant, *i.e.* *N*_*e*_ = (*N*_*e*, *i*_)_1:*M*_ and *γ* = (*γ*_*i*_)_1:*M*_, we prove that the models introduced in this section possess this important property.

**Proposition 1** (Identifiability of the nonparametric model, Eq (4)). *Let **g***^*A*^ *be distributed as a coalescent with EPS* (*N*_*e*, *i*_)_1:*M*_, *and* **g**^*B*^ *as a coalescent with EPS* (*γ*_*i*_*N*_*e*, *i*_)_1:*M*_, *M* ≥ 1, *then the vector* (*N*_*e*,1_, …, *N*_*e*, *M*_, *γ*_1_, …, *γ*_*M*_) *is identifiable*.

**Proposition 2** (Identifiability of the parametric model, Eq (5)). *Let **g***^*A*^ *be distributed as a coalescent with EPS* (*N*_*e*, *i*_)_1:*M*_, *and* **g**^*B*^ *as a coalescent with EPS* ${\left(\alpha N_{e,i}^{\beta }\right)}_{1:M}$, *if* *M* = 2, *the vector* (*N*_*e*,1_, …, *N*_*e*, *M*_, *α*, *β*) *is identifiable*.

Proofs of Propositions 1 and 2 can be found at Section A in S1 Text. [39] proves identifiability of *N*_*e*_ for the standard coalescent employing results in [35], which consists in showing that the expected Fisher information is non-singular. We follow the same template.

Similar to [39], we require for Propositions 1 and 2 to hold at least one coalescent event within each interval in the grid because at least one data point is needed to have non-zero Fisher information. This is not specific to our setting; it is also true in the classical case with a single population [39] when using a skyline estimator [40]. However, this is a theoretical result that takes into account only the likelihood. Our method is quite different because we employ a Bayesian formulation with GMRFs priors that add “structure” to the estimation problem in the sense of enforcing smooth estimates. This is likely to alleviate the requirements of at least one coalescent event per grid interval as evidenced by the empirical success of GMRF [13–16]. The reason is that the extra information carried in the prior helps addressing the lack of observations in a given interval.

### Extension to multiple populations

**Fig 2:**
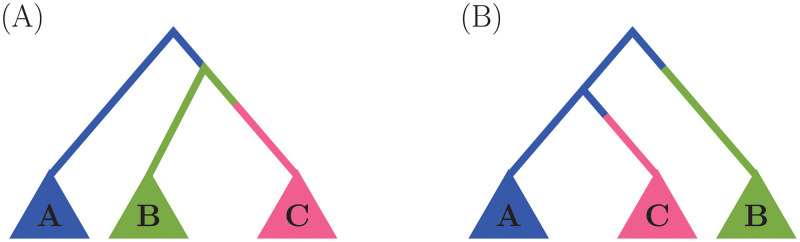
Modeling of multiple subpopulations with dependent population size dynamics. The trees represent large genealogies of many sequences from three different subpopulations labeled “A”, “B” and “C” at the tips of the trees. Each lineage represents the subtrees of individuals whose rate of coalescence is dictated by the color of the branch. (A) Nested populations. The blue branch indicates coalescent events happen at rate that depends on $N_{e}^{A}=N_{e}$, green branch indicates a coalescent rate that depends on $N_{e}^{B}=\gamma_{1}N_{e}$, and pink branch indicates a coalescent rate that depends on $N_{e}^{C}=\gamma_{1}\gamma_{2}N_{e}$. (B) Radial populations. The blue branch indicates a coalescent rate that depends on $N_{e}^{A}=N_{e}$, the green branch indicates a coalescent rate that depends on $N_{e}^{B}=\gamma_{1}N_{e}$, and pink branch indicates a coalescent rate that depends on $N_{e}^{C}=\gamma_{2}N_{e}$.

Our work is centered on a two-population model because one of our main applications targets the estimation of a relative advantage of a newly emerging viral variant over an existing variant. We further assume that the new variant originated from the standing variant. However, the framework can easily be extended to include multiple populations viewing the two-population model as a building block for a general genealogical model with multiple populations. In this generalization, the EPS of a child population is a function of the EPS of its parental population. This gives the two types of hierarchical structures displayed in Fig 2:

- *Nested populations.* Each effective population is a function of its immediate preceding one (Fig 2A). The baseline population “A” with EPS $N_{e}^{A}=N_{e}$ evolves into a second population “B” with EPS $N_{e}^{B}=\gamma_{1}N_{e}$, which then in turn evolves into a population “C” with EPS $N_{e}^{C}=\gamma_{1}\gamma_{2}N_{e}$.
- *Radial populations.* Multiple populations evolve from the baseline population “A” with EPS $N_{e}^{A}=N_{e}$ (Fig 2B). Then, population “B” will have EPS $N_{e}^{B}=\gamma_{1}N_{e}$, and population “C” with $N_{e}^{C}=\gamma_{2}N_{e}$.

The hierarchical structure for EPSs of multiple populations can be constructed iteratively as combinations of the two base structures of Fig 2. This construction maintains parameter identifiability. The inference framework for the multiple-populations extension still follows the Inference section: GMRF priors on the base EPS *N*_*e*_, and the *γ*_*i*_ parameters and the parameter inference is performed with INLA approximations. While we expect many evolutionary scenarios can be expressed in terms of the above hierarchical structures, if the underlying evolutionary process deviates from our model assumption, the model misspecification will lead to bias; formal investigation remains subject to further study.

Although Fig 2 may be interpreted as a realization of a multitype birth-death (MTBD) process [41], our model differs from the approach of MTBD in two important aspects. First, in modeling different genealogical processes, we assume a coalescent process governed by EPS, while MTBD assumes a branching process governed by birth, death, and sampling rates. Second, while both approaches aim to estimate subpopulation rates (EPS in our case, birth/death in MTBD), MTBD additionally targets the locations of the rate changes, whereas our model centers on how the rates of different subpopulations are related to each other over time.

## Results

We show the effectiveness of our methodology by applying it to synthetic and real-world data. In the synthetic data section, we offer evidence of its numerical accuracy. The real-data section illustrates the scientific insights that can be obtained by applying our methodology to SARS-CoV-2 data. The code to reproduce the simulation study is available at https://github.com/lorenzocapp/adapop_numexp.

### Synthetic data

**Fig 3:**
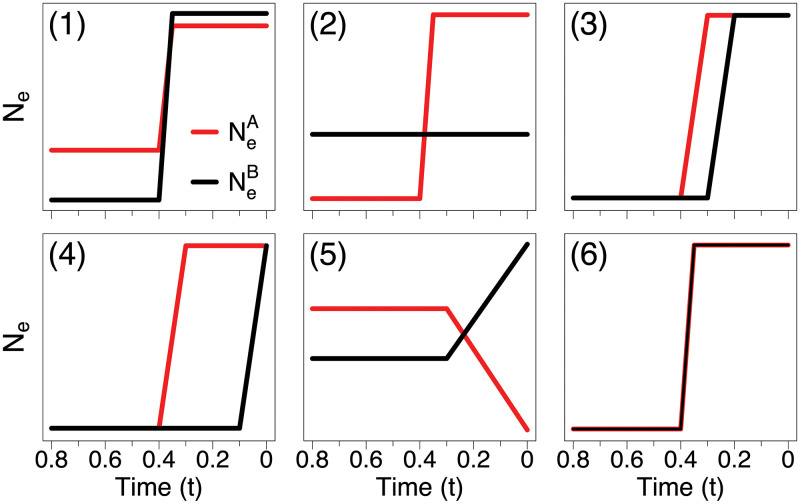
Population trajectories for synthetic data.

Fig 3 depicts six pairs of $\left(N_{e}^{A},N_{e}^{B}\right)$ used to simulate data. The trajectories mimic realistic scenarios typically encountered in applications, such as constant population sizes and exponential growths. The scenarios include several types of dependence between $N_{e}^{A}$ and $N_{e}^{B}$, ranging from perfect association (Scenario 6) to no association (Scenario 2). We simulated 100 datasets per each scenario. For a fixed pair of EPSs, we sampled (**s**^*A*^, **s**^*B*^), (**n**^*A*^, **n**^*B*^), and (**t**^*A*^, **t**^*B*^) with *n*^*A*^ = *n*^*B*^ = 200. Specifics of $\left(N_{e}^{A},N_{e}^{B}\right)$ and the data-generating mechanism can be found at Sections B–D in S1 Text.

We refer our hierarchical approach with and without preferential sampling as “adaPop” (Eq 4) and “adaPop+Pref” (Eq 6), respectively, and compare them to the parametric method (Eq 5) referred here as “parPop”. We also include a neutral estimator “noPop”, which ignores the association between populations and estimates $N_{e}^{A}$ and $N_{e}^{B}$ independently. We compare how accurately the four methodologies estimate *γ*, $N_{e}^{A}$ and $N_{e}^{B}$. Note that, while parPop and noPop do not approximate *γ*, the posterior *P*(*γ*|**s**^*A*^, **s**^*B*^, **t**^*A*^, **t**^*B*^) can be empirically approximated by taking samples from $P(N_{e}^{A},N_{e}^{B}|s^{A},s^{B},t^{A},t^{B})$ and summarizing $\gamma =N_{e}^{B}/N_{e}^{A}$. Let *f* be either *γ*, $N_{e}^{A}$ or $N_{e}^{B}$. We evaluate the performance of the methods using three metrics (listed below) computed on a regular grid of time points (*v*_*i*_)_1:*K*_. Here, the grid is defined with *K* = 100 on the interval [0, 0.6 *T*_*MRCA*_], where *T*_*MRCA*_ denotes the time to the most recent common ancestor at the root.

-
$\text{DEV}=\sum_{i=1}^{K}\frac{|\hat{f}\left(v_{i}\right)-f(v_{i})|}{f(v_{i})}$ , where $\hat{f}\left(v_{i}\right)$ is the posterior median of *f* at time *v*_*i*_. It is a measure of bias.
-
$\text{RWD}=\sum_{i=1}^{K}\frac{|{\hat{f}}_{97.5}\left(v_{i}\right)-{\hat{f}}_{2.5}\left(v_{i}\right)|}{f(v_{i})}$ , where ${\hat{f}}_{97.5}\left(v_{i}\right)$ and ${\hat{f}}_{2.5}\left(v_{i}\right)$ are respectively the 97.5% and 2.5% quantiles of the posterior distribution of *f*(*v*_*i*_). It describes the average width of the credible region.
-
$\text{ENV}=\sum_{i=1}^{K}1_{\left\{{\hat{f}}_{2.5}\left(v_{i}\right)\leq f\left(v_{i}\right)\leq {\hat{f}}_{97.5}\left(v_{i}\right)\right\}}$ . It is a measure of 95% credible intervals coverage.

Table 1 reports the average value of each statistic pooling together all datasets, all scenarios, and all grid points. Hence, each entry should represent the average performance of a method across the variety of challenging scenarios considered. We also average the performance metrics of $N_{e}^{A}$ and $N_{e}^{B}$. A more granular view of the performance of each method by scenario is given in Table A in S1 Text.

**Table 1:** Summary statistics of posterior inference of *γ*, $N_{e}^{A}$, and $N_{e}^{B}$. Each entry is computed as the mean of the performance metrics considered across all synthetic datasets: six possible scenarios for $\left(N_{e}^{A},N_{e}^{B}\right)$, and 100 datasets per a scenario. The metrics for $N_{e}^{A}$ and $N_{e}^{B}$ have also been averaged. Numbers in parentheses are the standard deviation of each estimate. The numbers in bold indicate the method(s) with the best performance (and within 10% of the best) for each performance metric: the highest for ENV and the lowest for DEV and RWD.

METHOD	$\bar{\text{ENV}}\gamma$	$\bar{\text{DEV}}\gamma$	$\bar{\text{RWD}}\gamma$	$\bar{\text{ENV}}N_{e}$	$\bar{\text{DEV}}N_{e}$	$\bar{\text{RWD}}N_{e}$
adaPop+Pref	**0.89**	**0.34**	**4.41**	**0.93**	**0.3**	**3.15**
	(0.12)	(0.21)	(15.71)	(0.08)	(0.27)	(13.58)
adaPop	**0.93**	**0.36**	6.96	**0.95**	0.39	5.65
	(0.1)	(0.24)	(26.92)	(0.06)	(0.37)	(25.04)
noPop	**0.94**	0.52	90.67	**0.95**	0.34	6.27
	(0.09)	(0.43)	(1144.69)	(0.07)	(0.28)	(24.08)
parPop	0.74	0.55	29.38	0.77	1.03	27.53
	(0.28)	(0.41)	(305.45)	(0.29)	(6.81)	(673.73)

adaPop+Pref stands out as the best performing method, exhibiting the lowest bias (DEV) and the narrowest credible regions (RWD). The coverage (ENV) is slightly worse than noPop; however, noPop achieves the slightly higher coverage with much wider credible regions. adaPop has a very similar performance to adaPop+Pref.

Sampling times were sampled at uniform and not proportionally to $N_{e}^{A}$ and $N_{e}^{B}$. This is the case where preferential sampling is less informative. Remarkably, adaPop+Pref still has narrower credible regions than adaPop. The reason is that the time-varying coefficient *ζ* is able to capture a variety of sampling protocols, including uniform sampling. We interpret this as an empirical evidence of the adaptivity of the model.

adaPop has narrower credible regions than the competing methodologies (noPop, parPop). This is an empirical proof of the “borrowing of information” of a hierarchical model. Notably, this property holds consistently across scenarios. adaPop’s higher $\bar{\text{DEV}}N_{e}$ is mostly attributed to poorer performance in estimating $N_{e}^{B}$ in Scenario 2 (see Table A in S1 Text). If we exclude Scenario 2, adaPop is superior to noPop and parPop across all metrics. Similarly, parPop is very competitive in the scenarios where the model is correctly specified (Scenarios 1, 2, and 6). The average performance deteriorates due to poorer performance in the remaining scenarios.

Lastly, an essential feature of adaPop and adaPop+Pref is that they are the most stable methodologies. This can be seen from the standard deviations of the statistics in the parentheses. Table A in S1 Text includes further analyses where the robustness and performance of the models are evaluated.

### Real data

Since its introduction, SARS-CoV-2 has undergone rapid evolution resulting in novel variants, some of which possess transmissibility, pathogenicity, or antigenicity advantages over the preexisting resident variants [42, 43]. A variant of recent interest is the delta variant (Pango lineage B.1.617.2 and AY lineages [44]). We compare this variant to other SARS-CoV-2 variants in two countries, South Korea and Italy.

We analyzed high-coverage complete sequences publicly available in GISAID [45] collected from South Korea and Italy during 2021-03-01 to 2021-09-30. For each country, we subsampled two sets of 150 sequences: one with the delta variant and the other without the delta variant. The details of the sequences used for our analysis can be found at Table B and Fig A in S1 Text. We then estimated the maximum credibility clade (MCC) trees—the tree in the posterior sample with the maximum sum of the posterior clade probabilities—of samples from each variant group of each country independently with BEAST2 [3]; the further analysis pipeline can be found at Section E in S1 Text. In our study, we set the population of the delta variant sequences as population *A* and of the non-delta variant as population *B*. Here, we discuss the results using the parPop and adaPop+Pref models. The additional results with other methods and further details of the sequence analysis pipeline, together with the inferred MCC trees, appear in Figs B–F in S1 Text.

**Fig 4:**
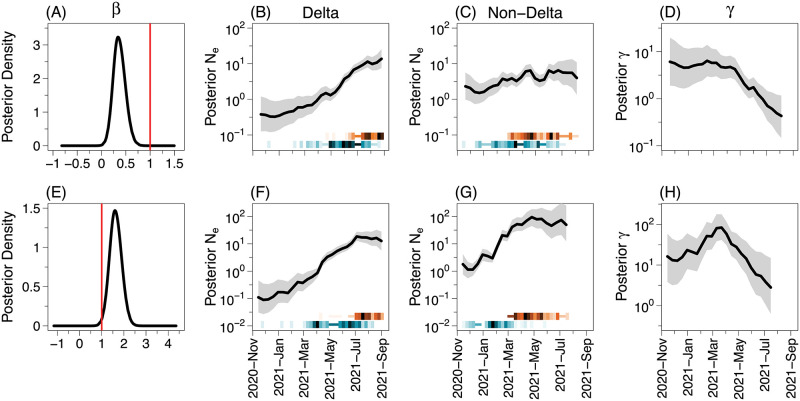
Posterior inference of SARS-CoV-2 population dynamics in South Korea and Italy. Panels A–D contain results of South Korea, and the panels E–H show results of Italy with **g**^*A*^=delta and **g**^*B*^=non-delta. The first column shows the *β* parameter posterior density from the parPop method. The red line indicates the value of *β* under the hypothesis that both variants share the same EPS trajectory in the parPop model. The other columns present the results with adaPop+Pref: the second and the third columns display posterior estimates of EPS of the delta and non-delta variant, respectively, and the last column shows posterior estimates of *γ*. The solid line indicates the posterior medians with its surrounding shaded areas representing 95% BCIs. The orange and blue heatmaps describe the sampling and coalescent event intensity, respectively: the darker the color, the more number of events occurs in a time interval. The *y*-axis of plots in the columns 2–4 is plotted on a log scale. The results using other methods can be found in the Figs D–F in S1 Text.

The orange-shaded heatmaps in Fig 4 depict the number of samples collected over time of the two populations in South Korea and Italy (as represented in our sub-sampled data sets). The observed pattern is consistent across the two countries: delta viral samples were predominantly collected in the summer of 2021, while non-delta samples were collected in the spring and early summer of 2021. This is consistent with the general observation of rapid spread of the delta variant that has progressively replaced the preexisting non-delta variants (such as alpha) since its introduction [46, 47].

Fig 4B, 4C, 4F and 4G depict the estimated posterior distribution of EPS of the two viral populations in the two countries obtained with adaPref+Pop (noPop estimates are qualitatively similar, see Fig D in S1 Text). In both countries, the delta population has experienced prolonged growth since its inception and halted its growth in the last month. On the other hand, the non-delta population EPS grew until approximately the end of May (the growth looks more pronounced in Italy), then it roughly plateaued.

We next examine the quantification of the dependence between the two populations: *β* for parPop and *γ* for adaPop+Pref. In Fig 4A, the posterior density of *β* estimated under the parPop model has mean 0.36 with 95% credible interval (0.19, 0.51), well below 1 (red line), suggesting EPS growth is more pronounced among sequences having the delta variant in South Korea. On the other hand, the posterior density of *β* has mean 1.62 with credible interval (1.25, 1.95) (Fig 4E) indicating the EPS of the non-delta variant grows faster than the delta variant in Italy under the parPop model. The result suggests that the growth of the non-delta population dominated that of the delta population which contradicts the general consensus [46, 47].

Under the adaPop+Pref model, the monotonic decrease in the growth of the non-delta variant EPS compared to that of the delta variant EPS in South Korea is apparent in *γ*-trajectory (Fig 4D); this is consistent with parPop *β*. However, the *γ*-trajectory of Italy (Fig 4H) shows that, compared to the growth of the delta variant EPS, the non-delta variant underwent an initial phase of faster growth and then transitioned to slower growth around mid-March of 2021. The inability of the parPop model (Fig 4E) to capture the two-phase population dynamics in Italy, that are evident in the adaPop+Pref model (Fig 4H), suggests that more flexible approaches proposed by our work are needed for accommodating the broad range of population dynamics scenarios encountered in real applications. We interpret the discrepancy between *β* and *γ* in this case as the evidence that parPop is not correctly specified.

## Discussion

We have developed a coalescent-based Bayesian methodology for inferring dependent population size trajectories and quantifying such dependency. We make minimal assumptions on the functional form of population size trajectories and allow the dependence between the two populations to vary over time. We also present a sampling-aware model for leveraging additional information contained in sampling times for reduced bias and improved inference accuracy. Although the proposed models have an increased number of parameters, we prove that the models are identifiable. We have shown that our adaPop+Pref outperforms other methods in synthetic data with known ground truth and that our adaptive method can detect changes in population size dynamics that are otherwise undetected with other models. We make this point more precise in our SARS-CoV-2 analyses.

We have decided to infer parameters via a numerically approximated method that relies on Laplace approximations (INLA) for computational speed. However, our proposed models can, in principle, be implemented in any of the MCMC standard approaches. Implementing our models in an MCMC approach would allow to infer population size trajectories from molecular sequences and sequencing time information directly and it is a subject of future development. This extension would account for uncertainty in the genealogy, an important component that is missing in the analyses presented.

We see a growing number of applications of the coalescent requiring the modeling of complex demographic histories. In the introduction, we mentioned a few, such as viral epidemiology and cancer evolution. There is extensive literature on models incorporating more and more realistic features, for example, a detailed description of migration histories. We note however that our proposed model explicitly models the dependency of population trajectories, providing a more interpretable dependency than in the structured coalescent. The quest for realism and scientifically meaningful parameters comes at the cost of computational tractability and leads, sometimes, to issues of model identifiability. Our work is somewhat motivated by these problems. Our method is equipped with high performance and accuracy, due to its scalability, interpretability, and parameter identifiability; such properties are lacking in many complex models in biology and epidemiology. We see our proposal as a “hybrid approach” that allows scientists to quantify the relative advantage of one population over another while still retaining a fairly parsimonious model. Such an approach will be invaluable across many biomedical disciplines for studying complex time-varying dependent evolutionary dynamics of populations.

## Supporting information

S1 Text**Section A. Proofs of identifiability. Section B. Simulation details. Section C. Grid construction. Section D. Synthetic data: additional results. Section E. SARS-CoV-2 molecular data analysis.** **Table A. Summary statistics of posterior inference of *γ*, $N_{e}^{A}$, and $N_{e}^{B}$.** Each entry is computed as the mean of the performance metric for a given scenario (100 datasets per scenario). The metrics for $N_{e}^{A}$ and $N_{e}^{B}$ have been also averaged. The numbers in bold indicate the method(s) with the best performance (and within 10% of the best) for each performance metric: the highest for ENV, the lowest for DEV and RWD. **Table B. GISAID EPI_SET IDs and their corresponding DOIs for sequences used in the real data analysis.** Each EPI_SET dataset contains 150 sequences. Note that EPI_ISL_402124 (hCoV-19/Wuhan/WIV04/2019, the official reference sequence employed by GISAID) is automatically included in the DOI EPI_SET web viewer generated by GISAID in addition to the 150 sequences per each dataset below. **Fig A. Collection date distributions of available high-coverage complete sequences in GISAID.** (A) South Korea. (B) Italy. Purple and green colors indicate sequences with and without delta variants, respectively. **Fig B. MCC trees of delta and non-delta variants from South Korea.** **Fig C. MCC trees of delta and non-delta variants from Italy.** **Fig D. Posterior EPS trajectories using the noPop method.** (A) South Korea, delta EPS. (B) South Korea, non-delta EPS. (C) Italy, delta EPS. (D) Italy, non-delta EPS. The figure format follows Fig 4 of the main text. **Fig E. Posterior densities of parameters and posterior EPS trajectories using the parPop method.** (A) South Korea, log *α*. (B) South Korea, *β*. (C) South Korea, delta EPS. (D) South Korea, non-delta EPS. (E) Italy, log *α*. (F) Italy, *β*. (G) Italy, delta EPS. (H) Italy, non-delta EPS. The figure format follows Fig 4 of the main text. **Fig F. Posterior EPS trajectories and posterior estimates of *γ* using the adaPop method.** (A) South Korea, delta EPS. (B) South Korea, non-delta EPS. (C) South Korea, *γ*, (D) Italy, *β*. (E) Italy, delta EPS. (F) Italy, *γ*. The figure format follows Fig 4 of the main text.(PDF)
